# Filter materials for metal removal from mine drainage—a review

**DOI:** 10.1007/s11356-014-2903-y

**Published:** 2014-05-01

**Authors:** Lena Johansson Westholm, Eveliina Repo, Mika Sillanpää

**Affiliations:** 1 School of Business, Society and Engineering, Mälardalen University, P.O. Box 883, 721 23 Västerås, Sweden; 2 Laboratory of Green Chemistry, LUT Savo Sustainable Technologies, Lappeenranta University of Technology, Sammonkatu 12, 50130 Mikkeli, Finland

**Keywords:** Organic materials, Inorganic materials, Adsorption, Metal removal, Normalisation, Mining wastewater

## Abstract

A large number of filter materials, organic and inorganic, for removal of heavy metals in mine drainage have been reviewed. Bark, chitin, chitosan, commercial ion exchangers, dairy manure compost, lignite, peat, rice husks, vegetal compost, and yeast are examples of organic materials, while bio-carbons, calcareous shale, dolomite, fly ash, limestone, olivine, steel slag materials and zeolites are examples of inorganic materials. The majority of these filter materials have been investigated in laboratory studies, based on various experimental set-ups (batch and/or column tests) and different conditions. A few materials, for instance steel slag materials, have also been subjects to field investigations under real-life conditions. The results from these investigations show that steel slag materials have the potential to remove heavy metals under different conditions. Ion exchange has been suggested as the major metal removal mechanisms not only for steel slag but also for lignite. Other suggested removal mechanisms have also been identified. Adsorption has been suggested important for activated carbon, precipitation for chitosan and sulphate reduction for olivine. General findings indicate that the results with regard to metal removal vary due to experimental set ups, composition of mine drainage and properties of filter materials and the discrepancies between studies renders normalisation of data difficult. However, the literature reveals that Fe, Zn, Pb, Hg and Al are removed to a large extent. Further investigations, especially under real-life conditions, are however necessary in order to find suitable filter materials for treatment of mine drainage.

## Introduction

Working and abandoned mines world over are continuously discharging mine drainage into surface and groundwater bodies (Dybrowska et al. 2006; Perez-Lopez et al. 2007; Chockalingam and Subramanian 2009; Potgieter-Vermaak et al. 2006; Strosnider and Nairn 2010; Trumm and Watts 2010; Prasad and Mortimer 2011; Goetz and Riefler 2014) and the worldwide mining industry is facing enormous challenges with the mine drainage. Mining effluents are characterised by high concentrations of heavy metals and high acidity, a combination that in many cases causes severe environmental problems such as acidification and lethal poisoning of aquatic organisms (Chockalingam and Subramanian 2009). Mine drainage can, however, be treated before being discharged into recipients, e.g. through active or passive treatment methods (Johnson and Hallberg 2005); thus, the mining industry has to rely upon some of these methods (Batty and Younger 2004). The former are based on the addition of chemicals and/or energy, while the latter are based on treatment systems such as wetlands, permeable reactive barriers, inorganic media passive systems, reducing and alkalinity producing systems and re-use of waste materials (Johnson and Hallberg 2005).

Passive treatment systems based on the flow of mine drainage through a filter material are advantageous in the sense, that they are regarded as low-cost solutions, thus interesting for the mining industry as well as for societies that have to deal with treatment of mine drainage. Scientists all over the world have therefore tested a large number of filter materials and scattered research has been presented on a wide variety of potential filter materials with regard to their metal sorption capacities in the first hand.

Natural materials, e.g. minerals, rocks or organic compounds, have together with various by-products from the industrial or agricultural sectors, gained particular attention as attractive filter materials for the removal of heavy metals and, in some cases, also as alkalinity providers. Tested filter materials have proved to remove heavy metals, and different metal removal mechanisms taking place in the filter materials have been identified, e.g. adsorption, ion exchange, sulphate reduction (Robinson-Lora and Brennan 2009) and precipitation (Feng et al. 2004; Rios et al. 2008). A variety of investigations, e.g. laboratory investigations as well as field trials, have been described in the literature.

Nonetheless, a compilation of data on the removal of heavy metals from mining wastewater using filter materials is lacking, even though there are a large number of reviews on filter materials and their capacities to remove heavy metals from wastewaters available, see for instance (Bailey et al. 1999; Wantanaphong et al. 2005; Nehdi and Tariq 2007; Ahmaruzzaman 2011; Iakovleva and Sillanpää 2013). The overall aim with this paper is therefore to give an overview of the literature on heavy metal removal from mining drainage by different substrates. Further on, the aims are to discuss a possible normalisation of results, as well as to discuss whether the filter materials are beneficial for on-site treatment of mine drainage. Finally, the paper also might serve as a tool to help others to select suitable filter materials based on the findings presented in the literature, e.g. metal reduction capacity, availability and cost.

## Mine drainage, removal mechanisms, filter materials and experimental methods

According to the literature reviewed, the filter materials can be divided into organic and inorganic materials. The first category includes materials such as peat and agricultural waste products. In addition, various organic polymeric materials have been investigated. The inorganic materials described in the literature include minerals and rocks and a variety of industrial waste products. These waste products have, from time to time, been deposited at landfill sites since there have been no use for them. At times when a shortage of natural resources has occurred, the industrial waste products have attracted attention as potential candidate materials for metal removal. Their potential to remove metals has just been one reason for the attention, in addition many filter materials a regarded as low-cost materials, easily available on a local scale. They are used in passive treatment systems that require a minimum of maintenance, which is another advantage compared to active treatment systems that might be in need of much maintenance as well as input of chemicals and/or energy (Johnson and Hallberg 2005). In this survey, steel slag materials and different types of ashes have been identified as potential filter materials for metal removal from mine drainage.

### Mine drainage

Table 1 presents a variety of mine drainages that have been studied in the literature. From the table it can be seen that pH values vary depending on the elements mined and conditions at the mining sites. It has been suggested that mine wastewaters could be divided in to three categories according to their acid/base properties (Iakovleva and Sillanpää 2013). These categories are acid mine drainage (AMD), neutral mine drainage and saline mine drainage. Acid mine drainage is characterised by pH values at 6 or below. This type of mine drainage occurs at sites where the rock is rich in sulphide minerals. Neutral mine drainage with pH values above 6 occurs where the rock is less abundant with regard to sulphides. Finally, saline mine drainage, characterised by pH values below 6 in combination with a salinity of 1,000 mgL^−1^ carbonates, are commonly found at locations where saline minerals are in abundance. Many researchers regard acid mine drainage as the major problem of the mining industry due to its toxicity.

**Table 1:** Compositions of real mining effluents studied (mg/L)

	Cu	Ni	Mn	Hg	As	Pb	Zn	Fe	Al	Au	Cd	Co	U	CN	SO_4_	Cl	pH	Reference
Clear Creek, AMD	0	0.01	0.97	–	–	–	0.32	–	–	–	–	–	–	–	–	–	–	Shin et al. 2008
Coal washing plant Mpumalanga, AMD	7.1	–	96	–	–	0.46	16	6,600	450	–	–	–	–	–	25,000	370	2.8	Gitari et al. 2008
South African gold mine, AMD	4.7	–	140	–	–	4.7	17	760	350	1.11	–	–	–	–	5,200	670	–	Feng et al. 2004
Nigel Town, ME	8	2	28	–	6	–	–	3	–	0.3	–	–	–	–	–	–	–	Mamba et al. 2009
Santa Rosa gold mine, ME	5.4	–	–	0.035	0.04	0.34	0.23	2.1	1.8	–	–	–	–	73	–	–	9.0	Benavente et al. 2011
Sideropolis coal mine, AMD	0.2	–	–	–	–	–	–	110	70	–	–	–	–	–	–	–	2.6	Laus et al. 2007
Uranium mine Brazil, ME	–	–	170	–	–	–	41.0	180	170	–	–	–	12	–	1,400	–	2.7	Ladeira and Goncalves 2007
Gornyak waste pile (old), Russia, AMD	37	0.6	–	–	–	0.4	160	210	350	–	0.86	0.8	–	–	–	–	2.7	Bogush and Voronin 2011
Gornyak waste pile (new), Russia, AMD	20	–	–	–	–	0.56	94	9.3	77	–	0.46	–	–	–	–	–	3.4	Bogush and Voronin 2011
Belovo sludge pond, Russia, AMD	730	5.9	–	–	–	–	910	0.18	21	–	6.0	5.1	–	–	–	–	4.1	Bogush and Voronin 2011
Mine leachate from oxidised tailings, Mexico	7.4	–	–	–	<0.002	0.01	310	40	–	–	3.58	–	–	–	2,500	–	2.7	Romero et al. 2011
Mine leachate from underground mine, Mexico	16	–	–	–	0.65	1.8	860	500	–	–	7.0	–	–	–	5,500	–	2.5	Romero et al. 2011
Beech Creek, AMD	–	–	5.8	–	–	–	–	9.3	1.7	–	–	–	–	–	390	8.9	3.5	Robinson-Lora and Brennan 2009
North Fork, AMD	–	–	3.5	–	–	–	–	7.4	2.9	–	–	–	–	–	180	15	3.5	Robinson-Lora and Brennan 2009
Cherry Run, AMD	–	–	2.3	–	–	–	–	1.2	1.6	–	–	–	–	–	290	6.2	3.3	Robinson-Lora and Brennan 2009
Kittaning Run, AMD	–	–	15	–	–	–	–	10	10	–	–	–	–	–	570	–	3.0	Robinson-Lora and Brennan 2009
AMD from Mpumalanga Province, South Africa	–	7 ± 0.2	230 ± 5	–	–	–	49 ± 31	5,600 ± 80	1,100 ± 10	–	–	4.3 ± 0.11	–	–	12,000 ± 20	–	2.4 ± 0.05	Vadapalli et al. 2012

*ME* mining effluents

### Removal mechanisms

Depending on the characteristics of the mine drainage as described above, different metal removal mechanisms have been suggested. Adsorption, ion exchange, sulphate reduction and precipitation have been identified as responsible mechanisms in various investigations. Adsorption, the adherence of a metal ion onto the surface of the solid filter material, can be strong or weak depending on physical or chemical sorption forces. Usually, adsorption takes place through functional groups existing on the adsorbent surface. For example, electrostatic interactions between cationic metals and anionic surface groups such as carboxyl have been suggested to contribute on the adsorption of metals by activated carbons (Mohan and Chander 2001). Equation (1) shows a possible reaction scheme between surface carboxyl groups and a metal ion (Chockalingam and Subramanian 2006):

1 $$ 2\kern0.5em {\mathrm{SCOO}}^{-}+{\mathrm{M}}^{2+}\to {\left(\mathrm{SCOO}\right)}_2\mathrm{M} $$

where S is a filter material, and M is a metal ion.

Ion exchange is based on the existence of electrically charged groups on the surface of the filter material. These can be changed with charged groups present in the surrounding solution and depending on the charge of the group; anion and cation exchangers are described. In the case of lignite and steel slag, Ca release occurs during sorption of target metals (Feng et al. 2004; Mohan and Chander 2006a). This ion-exchange mechanism can be written as:

2 $$ -\mathrm{SCa}+{\mathrm{M}}^{2+}\to -\mathrm{SM}+{\mathrm{Ca}}^{2+} $$

Quite often ion exchange and adsorption are difficult to separate. For instance, when carboxyl groups are protonated (see Eq. 1) ion exchange occurs between protons and metals.

Sulphate reduction involves bacteria and/or archaea living in oxygen-depleted environments. By oxidising hydrogen (H_2_) and reducing sulphate (SO_4_^2−^) to hydrogen sulphide (H_2_S), the organisms obtain energy through breathing sulphate rather than oxygen. The following equations can be used to present metal removal by sulphate reduction (Utgikar et al. 2000):

3 $$ \mathrm{Organic}\kern0.5em \mathrm{matter}+{\mathrm{SO}}_4^{2-}\to {\mathrm{HS}}^{-}+{\mathrm{HCO}}_3^{-} $$

4 $$ {\mathrm{M}}^{2+}+{\mathrm{H}\mathrm{S}}^{-}\to \mathrm{MS}\downarrow +{\mathrm{H}}^{+} $$

Sulphate reduction causes metals to precipitate as sulphide. Precipitation is a process, in which compounds that are not dissolved in solution falls out as a solid matrix. When olivine flour was used to treat the synthetic acidic mine water, precipitation of ferric iron hydroxide was observed to occur as follows (Kleiv and Thornhill 2004):

5 $$ {\mathrm{Fe}}^{2+}+\frac{1}{4}{\mathrm{O}}_2+{\mathrm{H}}^{+}\to {\mathrm{Fe}}^{3+}+\frac{1}{2}{\mathrm{H}}_2\mathrm{O} $$

6 $$ {\mathrm{Fe}}^{3+}+3{\mathrm{H}}_2\mathrm{O}\to \mathrm{Fe}{\left(\mathrm{OH}\right)}_{3\left(\mathrm{s}\right)}+3{\mathrm{H}}^{+} $$

In addition, precipitation of Mn as rhodochrosite or as other minerals was suggested to occur when synthetic mine impacted water was treated with chitosan. Surface characterisation of the filter material before and after adsorption, however, could not be used to verify the uptake mechanism (Robinson-Lora and Brennan 2011). On the other hand, Mn, Pb, Cu, and Cd have been suggested to precipitate as phosphates in the presence of chitin based on the calculations by Visual Minteq software.

It should be noted, that precise metal removal mechanisms are very difficult to determine. Usually, more than one mechanism is contributing in the metal removal at the same time especially in the case of low-cost filter materials with variable compositions. Therefore, it is out of the scope of this review paper to present any more details related to this issue.

### Filter materials

#### Organic materials

Table 2 presents organic filter materials as well as information on the type of experiment and main results from the various studies described in the literature review.

**Table 2:** Presentation of organic filter materials investigated with regard to metal removal

Filter material	Description of filter material	Character of experiment	Experimental conditions	Sorption capacity and other main results	Reference
Bark	Fresh bark from *Eucalyptus tereticornis* was ground (44 mesh)	Laboratory investigation	Batch tests using various amounts of filter material and AMD agitated for various intervals of time. Calculation of adsorption isotherms	Metal removal for Fe, Zn and Cu were reported to be 96, 75 and 92 % respectively	Chockalingam and Subramanian 2009
Chitin	Chitorem SC-20 Chitorem SC-80	Laboratory investigation	Batch studies: different doses of SC-20 was added into spiked mining influenced water and mixed for 24 h Adsorption by SC-80 studied in single-metal solutions. Effect of pH and contact time was also tested	Removal efficiencies: > 99.8 % Fe (120 mg/L), Pb (1.1 mg/L) and Zn (79 mg/L) 96 % Cd (1.3 mg/L), 54 % Co (0.78 mg/L), 42 % Cu (72 mg/L), 64 % Mn (52 mg/L)	Pinto et al. 2011
	Chitorem SC-20 Chitorem SC-40 Chitorem SC-80	Laboratory investigation	0.25 g of chitin was mixed with 0.1 L of AMD obtained from an abandoned coal mine. Samples were taken periodically	99 % removal of Al (10.9 mg/L), Fe (18.9 mg/L) and Zn (0.59 mg/L) and 98 % removal of Mn (19.8 mg/L)	Korte et al. 2008
	Chitorem SC-20	Laboratory investigation	Batch and column studies were conducted for different AMD samples. Pore volume of the column : 540 mL, flow rate: 0.25 mL/min, 25 g of SC-20	Complete removal of Al (1.6–10 mg/L), Fe (1.2–10 mg/L), and Mn (2.3–15 mg/L) for 171 pore volumes	Robinson-Lora and Brennan 2009
Chitosan	Chitosan extracted from shrimp waste	Laboratory investigation	Chitosan was mixed with gold ore tailing solutions. pH, equilibrium and kinetic studies were performed using synthetic wastewaters	Removal efficiency ranged between: 97 and 97.8 % for Cu (0.34–5.36 mg/L), 90–94.1 % for Pb 1.7–35.4), 95–98 % for Hg (0.07–0.23), 71.3–85 % for Zn	Benavente et al. 2011
	Chitosan microspheres	Laboratory investigation	2–30 g/L of chitosan microspheres were added into coal mining effluents	Complete removal of Fe (112–446 mg/L), Al (66–136 mg/L), and Cu (0.2–0.6 mg/L)	Laus et al. 2007
Commercial ion exchangers	Lewatit MP-500	Laboratory investigation	Batch studies: synthetic wastewater	Capacity of Cu: 25 mg/mL of resin	Bachiller et al. 2004
	IRA 910U Dowex A	Laboratory investigation	Batch studies: 0.15–1 g/L of resin mixed with the real AMD. Column studies: 5 mL of resin packed in a glass column, flow rate 2 mL/min	Uptakes: 66–108 mg/g for IRA 910U and 53–79 mg/g for Dowex A. Breakthrough of U at around 600 bed volumes	Ladeira and Goncalves 2007
Dairy manure compost	Material obtained from a farm in Weifang city, China	Laboratory investigation	Batch experiments using simulated AMDs. Single and multi-metal systems. Effect of pH, ionic strength, initial metal concentration, and adsorption time studied	Adsorption capacities for single metal systems: Pb: 046, Cu: 0.428, and Zn 0.237 mmol/g. Ionic strength and competing conditions affected mostly on the Zn adsorption. Desorption of all metals was conducted successfully by 0.1 M HCl.	Zhang 2011
Lignite	Lignite samples from Martin Lake, TX, USA. Samples were powdered in the laboratory to minus 325 B.S.S mesh	Laboratory investigation	Batch experiments, investigated metals were Fe^2+^, Mn^2+^ and Fe^3+^ in single-component systems. Ca ions were added in multi-component systems. Experiments carried out at different temperatures, particle sizes, pHs and solid/liquid ratios	Removal capacities were 34.22, 11.90 and 28.54 mg/g for Fe^2+^, Fe^3+^ and Mn^2+^. Metal removal higher with increasing temperature for Fe^2+^, but lower for Mn^2+^. Ion exchange suggested as the major removal mechanism	Mohan and Chander 2006a
	Lignite samples from Martin Lake, TX, USA. Samples were powdered in the laboratory to minus 325 B.S.S mesh	Laboratory investigation	Column studies under down flow mode in single and multi-columns. Metal solutions containing Fe^2+^, Mn^2+^ and Fe^3+^ were used. Studies performed at different pHs	Removal of metals was almost 100 %	Mohan and Chander 2006b
Peat	Peat humic agent (PHA)	Laboratory investigation	Drainage water from abandoned mines was added to glass beakers and mixed with PHA in ratios 1:1,000, 1:500 and 1:100	Purification efficiency ranged between 21 and 95 % for Fe, 17–99.9 % for Al, 11–99.9 % for Zn, 8–99.9 % for Cu, 8–99.9 % for Cd, 98 % for Pb, 3–95 % for Ni and 5–94 % for Co	Bogush and Voronin 2011
Rice husk	Rice husk (*d*_50_ size 206 μm and surface area of 0.68 m^2^/g) from rice mill in Bangalore, India was used.	Laboratory investigation	Rice husk and AMD (varying ratios) were mixed and agitated at 30 °C for different intervals of time	Almost complete removal of Fe^2+^, Fe^3+^, Zn^2+^ and Cu^2+^ at pH 3	Chockalingam and Subramanian 2006
Vegetal compost	Mixture of forest wood and sludge (9:1)	Laboratory investigation	Standard batch equilibrium experiments of prepared metal solutions mixed with vegetal compost	Metal loading of compost increased with pH and compost dose	Gibert et al. 2005
Yeast	*S. cerevisiae* samples from brewery industry (Seville, Spain)	Laboratory investigation	Yeast was mixed with synthetic, real and spiked AMD samples. Effect of contact time and metal concentration was studied	Adsorption efficiencies: >80 % for Cu (0.5 mg/L), 3.2 % for Mn (3 mg/L), 40 % for Ni (0.5 mg/L), 0 % for Zn (3 mg/L). Higher adsorption efficiency was obtained for Mn and Zn from synthetic wastewater; matrix did not affect adsorption of Cu and Ni	Ramirez-Paredes et al. 2011

Bark from *Eucalyptus tereticornis* (Smith) has, together with *Desulfotomaculum nigrificans*, been tested for its capacity to remove metals and sulphate from AMD (Chockalingam and Subramanian 2009). Fresh bark samples were washed thoroughly and dried before being ground and sieved (44 mesh). Acid mine drainage was collected at an abandoned pyrite mine pit in the northern Chitradurga district of Karnataka, India. In one experiment, 10 g of *E. tereticornis* was agitated with 100 mL AMD for different intervals of time; in another experiment the quantity of *E. tereticornis* varied keeping the time interval constant. Both set of experiments were carried out at a temperature of 30 °C. Adsorption isotherms were investigated as well. Chockalingam and Subramanian (2009) observed that approximately 96 % of Fe, 75 % of Zn, 92 % of Cu and 41 % of sulphate were removed from the AMD at pH 2.3. In addition, the pH value increased by two units after interaction with the bark material.

Chitin is the second abundant biopolymer in nature after cellulose. For commercial use, it is mainly obtained from crab and shrimp cells. Efficient removal of metals and dye molecules have been observed using chitin as a sorption material (Bhatnagar and Sillanpaa 2009). Chitin products with different purities and therefore different sorption capabilities are available. For instance, Chitorem SC-20 (40 % of CaCO_3_, 30 % protein, 20 % chitin and 3 % ash) and Chitorem SC-80 (88 % chitin and 12 % moisture) were used in the water treatment studies by (Pinto et al. 2011). 2 g/L of SC-20 removed almost completely Fe, Cd, Pb and Zn ions from the mining influenced water (Fe, 120; Cd, 1.3; Pb, 1.1; and Zn, 79 mg/L) collected from a mine in south-western USA. Moreover, 40–65 % of Co, Cu and Mn was removed (Co, 0.78; Cu, 72; and Mn, 52 mg/L) from the same effluent. Most of the metals were precipitated due to the presence of CaCO_3_ and hence increased alkalinity of the treated water. Some adsorption occurred, which was verified by the adsorption capacities of Pb (1.24 mg/g), Cd (1.81 mg/g), and Co (0.93 mg/g) on SC-80 in non-alkaline conditions (pH < 7). In addition, Korte et al. (2008) tested three different purities of chitin and observed the best metal adsorption performance for SC-20 from the mine impacted water sample collected from an abandoned coal mine in Pennsylvania. Both precipitation and adsorption was assigned to involve in the removal process. Protein part of SC-20 was observed to release NH_4_ in the solution fastening pH increase along with the dissolution of CaCO_3_.

In another study (Robinson-Lora and Brennan 2009), SC-20 was used in batch and column tests to neutralise and purify real AMD samples collected from different sites in North central Pennsylvania. A complete metal removal (Al, 10 mg/L; Fe, 10 mg/L; Mn, 15 mg/L) was obtained for 171 pore volumes and significant sulphate (570 mg/L) reduction (50–70 %) for 100 pore volumes (pore volume of the column, 540 mL; flow rate, 0.25 mL/min; 25 g of SC-20). Results indicated that metals were mainly precipitated as hydroxides, sulphides and carbonates. The same research group compared the remediation properties of chitin to commonly used lactate and spent mushroom compost in mining influenced water obtained from Kittaning Run in Altoona (Robinson-Lora and Brennan 2010b). They observed that chitin alone could work as effective neutralising agent, activator for sulphate reducing bacteria, as well as facilitator for metal (Fe, Al and Mn) removal, while lactate and mushroom compost needed at least some alkaline addition to function as efficiently. Moreover, chitin was the only substrate that could remove part of Mn likely due to rhodochrosite formation. Later on Robinson-Lora and Brennan (2010a) used demineralised and demineralised/deproteinised SC-20 for the manganese removal from a synthetic mine impacted water. Demineralised sample was consisted of chitin and its associated proteins, while its deproteinisation produced almost 100 % pure chitin. They observed that proteins played an important role in sorption phenomena giving over five times higher adsorption capacities for demineralised chitin compared to the almost protein free chitin. They also observed that bio sorption of Mn followed the Langmuir isotherm.

Chitosan is prepared from chitin by deacetylation. Metal removal properties of chitosan have been intensively studied (Varma et al. 2004; Guibal 2004). Recently, chitosan was tested for the heavy metal removal from the gold ore tailing solutions containing cyanide (Santa Rosa, Nicaragua) (Benavente et al. 2011). In these solutions, Cu (5.36 mg/L) for instance was mainly found as metal–cyanide complex, but still was removed nearly 98 % by chitosan. Coal mining effluents [decantation pool and AMD samples (Sideropolis, Brazil)] were treated successfully with chitosan microspheres (Laus et al. 2007). A chitosan dose of 15 g/L removed Fe, Al and Cu completely from the decantation pool sample (Fe, 446; Al, 136; and Cu, 0.6 mg/L; pH 2.34). Removal was assigned to both precipitation due to the increasing pH and complex formation due to the interactions between the amine groups of chitosan and metals.

Commercial ion exchangers are available for both anions and cat ions. A wide variety of ion exchangers with known properties is available. However, these have seldom been tested for the treatment of mining wastewaters. Lewatit MP-500 was used for the removal of copper cyanide complex from gold ore effluents, Spain (Bachiller et al. 2004). Copper recoveries were rather good, but the authors observed that presence of solids in the industrial effluent broke the particles of the resin decreasing its binding capacity during multiple cycles. Other researchers (Ladeira and Goncalves 2007) used IRA 910U and Dowex A for the removal of uranium from uranium mine wastewater, Brazil. Uranium breakthrough occurred at around 600 bed volumes for both ion exchangers. The presence of sulphate decreased the adsorption efficiency of uranium.

Dairy manure compost (DMC) containing abundantly lignin and chitin is generated large amounts especially in China. Due to its composition, DMC could be used as neutralising agent as well as biosorbent in the treatment of acidic mining wastewaters. The applicability of DMC from a Chinese farm for the removal of Pb, Cu and Zn from the synthetic AMD samples has been studied (Zhang 2011). Adsorption capacity of Pb was highest in both single and multi-metal systems, while the removal of Zn was mostly affected by the competing conditions and ionic strength. In addition, DMC was effectively regenerated by 0.1 HCl.

Lignite can be described as a low-grade coal; the coal content ranges from 25 to 35 %. Lignite is mined in different countries all over the world, and the major use is as fuel due to its rather high moisture content and low energy density. According to some researchers (Mohan and Chander 2006a), lignite possesses a high content of oxygen, which is fixed by carboxyl and hydroxyl groups. These groups act as important centres for ion exchange; thus, lignite materials can be used as cation exchangers. In a batch experiment, the sorption capacity of lignite samples from Texas, USA, was investigated (Mohan and Chander 2006a). Multi-component systems were used, and they concluded ion exchange to be the predominant sorption mechanism with regard to Fe^2+^, Fe^3+^ and Mn^2+^. They could also observe influence of the temperature on the sorption. Later on, Mohan and Chander (2006b) performed column studies using single and multi-columns set-ups. This research demonstrated that lignite can be used for the treatment of acid mine drainage contaminated with Fe^2+^, Fe^3+^ and Mn^2+^ also when interfering ions are present. Mohan and Chander (2006a) concluded that lignite can be applied for large-scale fixed bed reactors due to its removal capacity but also due to the high availability and low cost of the material.

Peat, partially decayed vegetation, is formed in wetland conditions and many different plant species can contribute to the composition even though the most common plant species are *Sphagnum* mosses. Peat has been used for water treatment purposes and also for treatment of AMD (Bogush and Voronin 2011). In a laboratory experiment, peat from Novosibirsk, Russia, was tested with regard to its metal sorption capacity. Three different drainage waters with different chemical compositions were used. Peat was added to the AMD in three ratios, e.g. 1:1,000, 1:500 and 1:100. The purification efficiency for the different waters/ratios were reported to range between 21 and 95 % for Fe, 17 and 99.9 % for Al, 11 and 99.9 % for Zn, 8 and 99.9 % for Cu, 8 and 99.9 % for Cd, 98 % for Pb, 3 and 95 % for Ni and 5 and 94 % for Co. The pH ranged from 2.9 to 8.1 after the experiment. The highest performance was, not surprisingly, obtained when increasing the peat/AMD ratio. It was concluded that peat is a good sorbent due to its high affinity for metal as well as a neutralising capacity (Bogush and Voronin 2011).

Rice husks are the hard protecting coverings of rice grains. They are made up of opaline silica and lignin, which protect the rice during the growing season. When the rice is harvested, the coverings are separated from the rice grains and since they are unsuitable for human consumption, other applications have been found, e.g., building materials, fertilizers, insulation materials, or fuel. Rice husk has also been investigated for treatment of AMD as a low-cost filter substrate in India (Chockalingam and Subramanian 2006). They investigated acid mine water from an abandoned mine and observed an almost complete removal of heavy metals (Fe^2+^, Fe^3+^, Zn^2+^ and Cu^2+^, studied. The high removal rate was explained by chemi-sorption.

Vegetal compost, e.g. the resulting product from aerobic composting of a mixture of forest woods and sludge at a ratio of 9:1, was studied with regard to its sorption capacity (Gibert et al. 2005). They performed laboratory experiments highlighting the sorption of Cu and Zn to develop a model for the prediction of their distribution in organic-based passive systems. Laboratory made metal solutions were used (metal concentrations ranged from 4 to 300 mg/L) and pH ranged from 2 to 6.5. Their results indicate that the metal loading of compost increased with pH and with the compost dose. Gibert et al. (2005) therefore concluded that there was a strong competition between hydrogen ions and metal ions for the available binding sites. In addition, they could conclude that their sorption data did contribute to the development of a model.

Yeasts are the by-products of the fermentation industry. Especially, *Saccharomyces cerevisiae* has shown a great potential as biosorbent for heavy metals due to its functionality, availability (easy cultivation, waste product), and low price (Wang and Chen 2006). Other researchers (Ramirez-Paredes et al. 2011) used *S. cerevisiae* (industrial bio-waste exhausted brewer’s yeast) for the metal removal from synthetic, real and spiked AMD samples. Results indicated that the removal of Cu and Ni was not affected by the matrix i.e. similar behaviour was observed in synthetic and real wastewater samples. However, real wastewater matrix clearly affected the removal of Mn and Zn, which was attributed to the antagonistic interference due to the presence of other metals in the solution. Generally, the removal process was associated to ion exchange, coordination, and complexation as well as hydrophobic, polar and van der Waals interactions. A slight increase in pH was also observed during the experiments.

##### Summary

A variety of organic filter materials have been investigated, the majority in laboratory experiments in which real-life or synthetic AMD were used. The results show that many of the filter materials removed metals to a large extent and different removal mechanisms were identified as well.

#### Inorganic materials

Table 3 presents inorganic filter materials that are described in the literature.

**Table 3:** Presentation of inorganic materials investigated with regard to metal removal

Filter material	Description of filter material	Character of experiment	Experimental conditions	Sorption capacity and other main results	Reference
Biocarbon	Prepared from pine by pyrolysis followed by steam activation. Specific surface area: 130–1,000 m^2^/g	Laboratory investigation	Batch tests by mixing biocarbon with two different AMD samples	Metal removal ranged between 10 and 95 %. Highest removal for Zn and Cu, lowest for Mn	Shin et al. 2008
Calcareous shale	Rock samples were dried and crushed to 10 mesh in the laboratory	Laboratory investigation	Batch tests using two mine leachates and one synthetic leachate mixed with filter material	Removal efficiency 100 % for As, Pb, Cu and Fe. Removal efficiency 87 % for Cd and 89 % for Zn.	Romero et al. 2011
Dolomite	Particle size <150 μm	Laboratory investigation	Jar Test. Laboratory prepared mine waters were mixed with dolomite in varying amounts for 6 h	Slightly more than 6 h contact time needed for 120–160 g dolomite/L to decrease the concentration of ferric ions to near 100 %	Potgieter-Vermaak et al. 2006
Fly ash	Untreated fly ash from a peat fired power station was used. Hydraulic conductivity 1.3 m/day, porosity 58 % and bulk density 0.83 t/m^3^.	Field investigation	Fly ash was mixed with sand in a small-scale treatment cell. Mine drainage passed through the cell with a residence time of approx.15 min. Operation period 2.5 weeks	Metal removal for Zn, Pb and Cd ranged between 98.6 and 99.9 %	Warrender et al. 2011
	Particle size <150 μm	Laboratory investigation	Jar Test. Laboratory prepared mine waters were mixed with fly ash in varying amounts for 6 h	6 h contact needed for 500 g fly ash/L to reduce levels of ferric ions to below 0.1 g/L (near complete removal)	Potgieter-Vermaak et al. 2006
	Petroleum coke fly ash from a combustion power plant in the Bío Bío region, Chile	Laboratory investigation	Batch and column tests. Batch leaching tests were performed in liquid :solid ratio of 10 L/kg with an agitation time of 24 h. Column tests were performed in small columns (height 0.1 m, diameter 0.05 m) at a flow rate of 0.2 mL/min.	Based on neutralization and heavy metal removal tests, one NCS tested could be suggested as a suitable sorbent. Maximum removal capacities observed for Cu^2+^ and Pb^2+^ were 8.1 and 28.3 mg/g respectively	González et al. 2011
	Fresh samples of fly ash from the Mpumalanga Province, South Africa	Laboratory investigation	Fly ash was added to AMD in beakers and stirred for 360 min. The mixture was allowed to settle before supernatant being decanted and analysed	Improved quality of supernatant when using high ratio AMD:fly ash was observed	Vadapalli et al. 2012
	Two different fly ashes from South Africa, both class F according to The American Society for Testing and Materials	Laboratory investigation	Raw AMD was filtered, diluted with MQ water and stabilised with HNO_3_ before being mixed with fly ash and stirred for 120–360 min. Two batch set-ups were performed	Removal of major and trace elements were high (>75 %). Final pH and amount fly ash were observed to be important for the removal. At optimal conditions nearly 100 % removal was observed	Gitari et al. 2006
	Fresh samples of fly ash from a coal combusting plant in South Africa	Laboratory investigation	Batch tests where fly ash and AMD was mixed in different ratios (1:3 and 1:1.5) for 1–1,440 min	Removal of metals was increased at ratio 1:1.5 due to suggested precipitation mechanisms. Other processes (adsorption and co-precipitation) were also suggested	Gitari et al. 2008
	Fly ash derived from a coal combustion in Los Barrios power plant, Cádiz, Spain	Laboratory investigation	Column tests where artificial irrigation of MQ water through a pyrite-rich residue resulted in a drainage solution similar to AMD low in pH, high in sulphate, iron and other heavy metals. Operation of columns was 30 weeks	Addition of fly ash to the pyrite-rich residue resulted in improvement of the drainage solution, metal immobilization and oxidation attenuation processes were effective and drainages were low in metal concentrations	Perez-Lopez et al. 2007
Limestone	Calcite limestone samples with two particle sizes (0.42–0.59 mm and <0.045 μm)	Laboratory investigation	Batch tests using particles size <0.045 μm. Fixed bed experiments using particle size 0.42–0.59 mm with flow rate ranging from 1 to 10 mL/min. Neutral mine water in both studies.	Langmuir isotherms showed a maximum uptake of sulphate of 23.7 mg/g limestone	Silva et al. 2012
	Limestone gravel from Karamea, New Zealand	Field experiment	Small-scale reducing and alkalinity producing systems (RAPS) treating two AMDs at two sites (Fe 10.6–47 mg/L, Al 1.6–14.1 mg/L and minor amounts of Mn, Ni and Zn) Limestone layer 1.3 × 0.56 × 0.13 m	The RAPS showed to be effective at removing metals. At site 1 Fe, Al and Ni were removed to 97, 100 and 66 % (residence time 5 h). Corresponding results for site 2 were 99, 96 and 95 % for Fe, Al and Ni	Trumm and Watts 2010
	–	Field experiment	Incubation regimes where AMD was mixed with domestic wastewater and incubated for 72 h.	Results showed that significant quantities of REE could be removed from solution	Strosnider and Nairn 2010
	Particle size <150 μm	Laboratory investigation	Jar Test. 40–160 g/L, contact time 240 min. Simulated AMD	Complete removal of ferric ions within 6 h	Potgieter-Vermaak et al. 2006
Olivine	Non-ferrous, forsterite olivine dust	Laboratory investigation	Laboratory-made Cu-solution (1.27–38.1 mg/L) used in batch adsorption experiments at 25 °C. Final pH 4–6	Results showed that the olivine dust greatly reduced Cu	Kleiv and Sandvik 2002
	Olivine flour (concentration of Forsterite 90%), specific surface area 4.3 m^2^/g	Laboratory investigation	Batch tests using a synthetic AMD (pH 3) containing Cu, Zn and various amounts of Fe.	The maximum retention of Cu was obtained at a solid/solution ratio of 10 g/L after 10 min contact time, 79 % reduction	Kleiv and Thornhill 2004
Steel slag materials	Washed iron making slag and steel making slag with mean particle sizes 24.5 μm and 24.1 μm respectively	Laboratory investigation	Batch sorption experiments using laboratory prepared metal (Cu and Pb) solutions were carried out at 18 °C. Contact time between slag and metal solution was 24 h	Saturation capacity of iron slag was 88.50 mg/g for Cu^2+^ and 95.24 mg/g for Pb^2+^. Saturation capacity of steel slag was 16.21 mg/g for Cu^2+^ and 32.26 mg/g for Pb^2+^	Feng et al. 2004
	Five types of steel mill wastes (BFS, oxygen gas sludge (OGS), evaporation cooler dust (ECD), electro-static precipitator dust (EPD) and BOFS) in the form of powder	Laboratory investigation	Batch tests were 1 g sorbent was mixed with 25 mL As-solution and stirred for 1–72 h before analysis	Results showed that ECD, OGS and BOFS effectively re-moved As from solution. EPD removed As to a lesser degree. In the BFS system, the As removal was enhanced at pH 6	Ahn et al. 2003
	A mix of BFS and BOF from a steel processing plant in South Wales was used. Hydr. cond. 13.9 m/day, porosity 43 % and bulk density 1.21 t/m^3^.	Field investigation	BFS and BOF were mixed in a small-scale treatment cell. Mine drainage passed through the cell with a residence time of approx.15 min. Operation period 2.5 weeks	Removal of Zn, Pb and Cd were 0.20, 0.08 and 0.0015 mg/g. The poor performance was attributed to the slags were weathered instead of fresh	Warrender et al. 2011
	Iron granules (0.6–2 mm) and GBFS (0.3–2 mm)	Laboratory investigation	Column (l 0.4 m, w 0.15 m) test using a real life AMD and a synthetic AMD with a flow rate of 30 mL/h	15 mg/L of As were reduced to less than 0.7 mg/L and 15 mg/L of Mn^2+^ ions were removed to less than detection limit using GBFS column.	Sasaki *et al*. 2008
	Steel slag	Field investigation	Steel slag and limestone beds were used for treatment of AMD at several locations in West Virginia, US. AMD was flowing through the passive bed systems that were constructed in 2000	Results indicated that metals found within the steel slag were immobile during the operation. Metal concentrations have remained low	Mack and Gutta 2009
	EAF steel slag	Field investigation	Steel slag leach bed systems were used for treatment of AMD in Raccoon Creek, southern Ohio, US. AMD was flowing through the passive bed systems for 2 years	The steel slag leach bed systems were reported to operate inconsistently and failure mechanisms were poorly understood	Kruse *et al*. 2012
	Steel slag	Field investigation	Steel slag leach beds were used for treatment of AMD in Mingo County, West Virginia, US. AMD from a surface coal mine flowed through the steel slag beds	Results indicated that steel slags can provide highly concentrated alkaline recharge to AMD over long periods	Ziemkiewicz 1998
Zeolites	Clinoptilolite exchanged with Fe^3+^	Laboratory investigation	Batch and column tests. 1.0 g of sorbent was mixed with 50 mL of As-solution (0.1–20 mg/L) for 24 h. In column tests, 400 g sorbent was packed in columns (height 0.7 m, diameter 0.05 m) and fed with AMD corresponding to 40 pore volumes	In batch tests, removal of As^3+^ was 100 mg/kg, and for As^5+^ 50 mg/kg. A complete As removal was obtained in column tests after 40 pore volumes	Li et al. 2011
	Clinoptilolite (1–3 mm)	Laboratory investigation	Batch tests at room temperature. 3.7, 7.5 and 15 g of sorbents was mixed with synthetic metal solutions for 15–360 min	About 80, 95, 90 and 99 % of Fe^3+^, Mn^2+^, Zn^2+^ and Cu^2+^ were removed during the first 40 min	Motsi et al. 2009
	Clinoptilolite (1–3 mm)	Laboratory investigation	Batch tests at room temperature. 3.7 g sorbent was mixed with 100 mL synthetic metal solution for 2–360 min	Intraparticle diffusion was observed to be the main rate controlling the removal of heavy metals	Motsi et al. 2011
	Synthetic zeolites (clinker-based faujasite)	Laboratory investigation	Batch tests carried out at room temperature. 20 mL of AMD was added to 0.25 or 1 g sorbent at initial pH 1.96 for 5 min to 24 h	Selectivity of faujasite for metal removal was Fe > As > Pb > Zn > Cu > Ni > Cr	Rios et al. 2008
	Synthetis zeolites (fly ash-based)	Laboratory investigation	Batch tests at room temperature. 50 mL of AMDs were mixed with zeolite powder (5.0–40 g/L) for 60 min	100, 98.9, 98.8, 85.6, 82.8, 48.3 and 44.8 % of Pb, Cd, Zn, Cu, Fe, Ni and Ba were removed	Prasad and Mortimer 2011

Bio-carbons are generally considered as low-cost alternatives for commercial activated carbons. The cost is reduced by utilising a locally available biomass in the preparation of bio-carbon adsorbent. Shin et al. (2008) used Lodgepole Pine as a starting material for bio-carbon. They applied moderate temperatures in pyrolysis (400 °C) following steam activation at 700 °C and obtained carbonous materials with high surface areas (130–1,000 m^2^/g). Initially, the pine was used as sawdust or cubes of different sizes, and the effect of pre-treatment of wooden material with KOH was also examined. Five different bio-carbons were then tested in the removal of metals from two different AMD samples obtained from Clear Creek and Leadville. Removal efficiencies were 18–79 % for Cd, 54–61 % for Cu, 7–35 % for Mn, 11–67 % for Ni, and 15–93 % for Zn from relatively diluted solutions (see Table 1). Adsorption efficiencies of bio-carbons were generally higher than obtained for the commercial activated carbon. It was also noticed that the surface area of the bio-carbon did not play a crucial role in adsorption.

Calcareous shale, an indigenous geological material in the Taxco Mining area in Mexico, was used for its metal removal and neutralisation capacities (Romero et al. 2011). They performed two batch experiments using crushed calcareous shale and solution, e.g. two types of acid mine leachates. Synthetic mine leachates was also added in the experiments. All leachates were composed of As, Pb, Cd, Cu, Fe and Zn. Sample suspensions were equilibrated for 18 h and continually shaken before being filtered. The results from the investigation showed that the removal efficiency of the calcareous shale was 100 % for As, Pb, Cu and Fe, while the removal efficiencies for Cd and Zn were 87 and 89 %, respectively. Romero et al. (2011) concluded that under laboratory-batch conditions, the calcareous shale was efficient with regard to metal removal. In addition, the calcareous shale contributed to the neutralisation of the mine leachates.

Dolomite, a rock-forming mineral mainly composed of CaMg(CO_3_)_2_ is found all over the world in vast deposits. Its main use is as an ornamental stone, a concrete aggregate and a source of magnesium oxide. Potgieter-Vermaak et al. (2006) compared dolomite, limestone and fly ash to lime in order to find out whether these agents would perform better and save costs when pre-treating acid mine drainage. These researchers used a Jar Test apparatus to investigate the influence of dolomite (and the other agents) on pH and the ion concentrations of different iron complexes, calcium, magnesium and sulphate. A simulated acid mine water was used for the investigation. The results indicated that the water quality did improve with increased amounts of dolomite and surface area, contact time and composition of the acid mine water. The dolomite (120–160 g/L) removed ferric ions to near completion in slightly more than 6 h. The dolomite was, however, outperformed by limestone that performed better in all aspects (Potgieter-Vermaak et al. 2006). In spite of this, Potgieter-Vermaak et al. (2006) concluded that dolomite could reduce costs if it could replace lime as a pre-treatment agent.

Fly ash is generated in combustion of coal, and it is composed of the fine particles that rise with the flue gases. Composition of fly ashes varies depending on source and composition of the coal being burned, but silicon dioxide (SiO_2_) and calcium oxide (CaO) are found in substantial amounts. Utilisation of fly ash ranges from its being a component in concrete products, embankments or as aggregate material in brick production. Fly ash has also been used as soil amender or fertilizer within the agriculture. Other applications include paints, cosmetics, and filler in wood and plastic products. It has also been used to transform sewage sludge into organic fertilizer or biofuel due to its alkalinity and water absorption capacity. Several researchers have investigated fly ash for treatment of mine drainage in recent years. Potgieter-Vermaak et al. (2006) investigated fly ash in the laboratory using a jar test apparatus. A simulated AMD was prepared, and in aliquots of 500 mL, the solution was placed in plastic beakers. The dosing agents, e.g. fly ash, were added and the solution was stirred for 30 min to 6 h. The results showed that 500 g/L fly ash removed ferrous ions in the AMD to very low levels as long as the contact time was 6 h. In a batch set-up, Gitari et al. (2006) carried out studies on removal of major contaminants and trace elements in AMD by South African fly ashes. Different ratios (fly ash/AMD) were used. In addition, pH varied as did the contact time. Gitari et al. (2006) observed that most elements were removed to nearly 100 % when the pH of minimum solubility of their hydroxides was achieved. Other factors influencing the removal of contaminants were the ratio fly ash/AMD and the contact time. In another study, Gitari et al. (2008) carried out batch experiments where fresh coal ash was sampled and mixed with AMD (ratio, 1:3 or 1:1.5). The mixtures were stirred for 1–1,440 min. The results from these investigations showed an increased removal of elements (Mg, Mn, Al, Si, tot-Fe, Zn and Cu) at the ratio of 1:1.5, indicating the importance of precipitation reactions taking place. Perez-Lopez et al. (2007) utilised column studies in order to study the process of acid neutralisation of AMD by fly ash. Once a week, Millipore MQ water was poured on the columns simulating an irrigation of approximately 1,040 L/m^2^ or the annual average of rainfall in the Andalucía region in Spain. The leachate from the columns, filled with sulphide-rich residues, was similar to an AMD with low pH, high concentrations of sulphate, iron and heavy metals. The addition of fly ash to the sulphide-rich residues improved the quality of the leachate from the columns since ferric hydroxide coatings formed on the surfaces efficiently immobilised toxic metals. In a field trial described by Warrender et al. (2011) fly ash from a peat fired power station was used in a small-scale passive treatment system for removal of Zn, Pb and Cd. Their results showed that 21.4, 0.88 and 0.04 mg/g of Zn, Pb and Cd, respectively, were removed.

Limestone is a sedimentary rock composed of calcite and aragonite, e.g. different crystal forms of calcium carbonate (CaCO_3_). Depending on genesis, limestone might also contain fragments of corals or foraminifera. There are different uses of limestone, e.g. for building and industrial purposes and it is also used for purification processes due to its reactive properties. Limestone reacts with sulphate, which is commonly occurring in mining wastewater where rocks containing sulphide minerals have been mined. Limestone has therefore been regarded as a neutraliser for AMD, particularly for mine waters that are neutral (Silva et al. 2012). Silva et al. (2012) performed batch and small-scale column tests where different mine waters were fed to a calcite limestone under various conditions (see Table 3). They found limestone to be a cost-effective alternative for treatment of mine waters with low concentration of sulphate. In addition, Potgieter-Vermaak et al. (2006) found limestone to be a feasible alternative to lime. Limestone was also used by Trumm and Watts (2010) who tested the media in small-scale pilot systems, e.g. as reducing and alkalinity producing systems, with regard to two acid mine waters in New Zealand. Trumm and Watts (2010) performed laboratory column tests as well as field trials and concluded that the small-scale systems might work as reducing system as well as an alkalinity producing system depending on the character of mine water. Trumm and Watts (2010) reported high removal rates. Strosnider and Nairn (2010) also investigated limestone in passive treatment systems for the removal of various elements; among them rare earth elements (REEs) found in some mine waters but usually not tracked according to the authors. In addition, Strosnider and Nairn (2010) co-treated the high-strength AMD with raw wastewater. The results from this study indicated that passive systems may remove other constituents than normally analysed, e.g. the REE. Further on, they suggested further studies on co-treatment of AMD and wastewater. In laboratory and field investigations, oxic limestone drains were investigated by Cravotta (2008). The reduction of Al, Fe and Mn was studied. The results showed that the metals were reduced but that the effectiveness of the treatment system declined with time, possibly due to clogging. Cravotta (2010) also studied limestone treatment in a variety of passive and semi-passive treatment systems to reduce the transport of heavy metals and to neutralise AMD in Pennsylvania. The results indicated that the wetlands effectively reduced the transport of metals as well as the acidity load. Cravotta (2010) therefore concluded that the systems had a positive effect on the environment, but that long-term studies were needed in order to follow the performance.

Olivine is one of the most abundant minerals in the Earths subsurface. It occurs as different members of the olivine solid solution series, and it is a magnesium iron silicate with the formula (MgFe)_2_SiO_4_. Olivine sand is used within the aluminium foundry industry to cast objects in aluminium in order to hold the mold together during handling and pouring of the metal. The non-ferrous olivine, forsterite olivine (Mg_2_·SiO_4_), has been investigated with regard to its metal removal capacity (Kleiv and Sandvik 2002). They considered forsterite olivine suitable as filter material since it constitutes both a neutralising agent as well as an adsorbent with high affinity for copper in particular. In a laboratory study by Kleiv and Thornhill (2004), forsterite olivine was subject to a synthetic mine water solution. The results from their investigation showed that the amount of Cu and pH were positively correlated with the solid: solution ratio. Further on, Kleiv and Thornhill (2004) observed a rapid increase in retention as a function of time if ferrous iron was present in the initial solution, but a drop in the retention with time was observed as well.

Steel slag materials are waste products from the steel making industry. Utilisation of steel slag is described by Shen and Forssberg (2003). Different steel slag materials, e.g. blast furnace slag (BFS), electric arc furnace slag (EAF), basic oxygen furnace slag (BOF), iron slag and steel making slag have been used for removal of metals from various wastewater streams such as landfill leachate and storm water (Nehrenheim et al. 2008). A few researchers have also used steel slag materials for removal of metals from acid mine drainage. Feng et al. (2004) investigated removal of Cu, Pb and Cr, and precious metals like Au, Ag, Pt, Pd, Rh and Ru present in acid mine drainage from an a South African gold mine, by iron slag and steel slag. The initial pH of the AMD was 2.03, but it increased to neutral as the slag dose increased up to 30 g/L. At this slag dose, most of the Cu, Pb and Cr were removed by the iron slag, which performed better than the steel slag. The higher surface area, higher porosity and higher ion-exchange ability of the iron slag were suggested to explain these results. The precious metals were only removed to a small extent, which was explained by their form as anionic complexes with chloride that not physically could adsorb onto the negatively charged slag surface. Mine drainage from a former Pb/Zn mine in Mid-Wales were used in small-scale passive treatment cells intended for removal of Cd, Zn and Pb (Warrender et al. 2011). A mixture of BFS and BOF was used as a filter material. The drainage water was circum-neutral pH (≈6.3), while the Fe concentrations were low (<0.2 mg/L). Concentrations of metals were elevated (≤30 mg/L Zn, ≤1 mg/L Pb and ≤0.1 mg/L Cd, respectively). The residential time of the filter material was approximately 15 min. The field experiment was run for a period of 2.5 weeks. The results showed the removal of 0.20, 0.08 and 0.0015 mg/g for Zn, Pb and Cd, respectively. Granulated blast furnace slag (GBFS) was used by Sasaki et al. (2008). They carried out a column experiment using a spiked AMD (pH 2.0, 50 mg/L Ca^2+^, 7.3 mg/L Na^+^, 60 mg/L Mg^2+^, 1,200 mg/L SO_4_^2−^, 30 mg/L Fe, 15 mg/L Mn and 15 mg/L As^5+^) and showed that a concentration of As decreased from 15 mg/L to <0.4 mg/L in the column performance.Jarvis and Younger (2001) report that BFS has successfully been used in a surface catalysed oxidation of ferrous iron (SCOOFI) system. In a batch experiment, Ahn et al. (2003) compared five different steel mill wastes, among them BOF and BFS, with regard to the removal of As^5+^ and As^3+^ in tailing leachate (pH non-controlled, 25 mg/L As^5+^ or 25 mg/L As^3+^). They reported that BOF effectively removed As^5+^ and As^3+^, while BFS did not remove As from solution at all. In the USA, several researchers (Simmons 2001; Simmons and Black 2002; Mack and Gutta 2009; Kruse et al. 2012; Ziemkiewicz 1998) have investigated steel slag as an alternative for limestone. Their objectives have been twofold, e.g. the metal removal has been investigated as well as the slag’s ability to supply alkalinity to streams receiving AMD. Steel slag has been used in open and/or underground mines. Several studies have been performed, both in the laboratory as well as in the field, and the common conclusion is that the steel slag removes heavy metal and in addition, it performs positively with regard to increasing the alkalinity in streams effected by AMD.

Zeolites are a group of naturally occurring alumina-silicates and comprise a large number of minerals. Zeolite deposits exist all over the world. It is also possible to synthesise zeolites. Common for all zeolites, natural as well as synthesised, is a highly porous structure, which make them suitable for adsorption. In addition, zeolites are also known to possess a high ion-exchange capacity. Zeolites are widely used for various industrial purposes such as water purification processes. A large number of researchers have investigated zeolites with regard to their capacities to remove pollutants, not only heavy metals, from various waste water streams. Different zeolites have been investigated according to the literature review; most tests have been carried out in laboratory investigations, and sorption capacities have been calculated. Motsi et al. (2009) performed batch experiments using clinoptilolite, a natural zeolite, in order to study the potential of the material to treat AMD containing Fe^3+^, Cu^2+^, Mn^2+^ and Zn^2+^. The adsorption rate as well as the uptake at equilibrium was studied using both single and multi-metal solutions. During the first 40 min of the experiments, approximately 80 % of the total adsorption occurred. After this rapid period, the adsorption decreased. From the equilibrium studies, Motsi et al. (2009) reported the selectivity sequence to be Fe^3+^ > Zn^2+^ > Cu^2+^ > Mn^2+^. In preliminary tests, AMD samples showed promising results, and Motsi et al. (2009) concluded natural zeolites to have a great potential as a low-cost material in the treatment of AMD. Later on, Motsi et al. (2011) carried out kinetic studies of the removal of the same heavy metals as investigated previously (Motsi et al. 2009). The kinetic studies revealed that intra-particle diffusion was the major step in the removal of heavy metals from solution by natural zeolite. Li et al. (2011) investigated removal of As from AMD using clinoptilolite exchanged with Fe^3+^ in batch and column tests. In the batch tests, in which a laboratory made As solution with varying concentrationswas used, the sorption capacity amounted to 100 mg/kg. In the column tests, AMD with an initial concentration of 147 μg/L As was fed to the columns resulting in a complete removal of the metal up to 40 pore volumes. Two variants of synthetic zeolites (similar to the faujasite type of zeolite) were used by Rios et al. (2008) who carried out batch experiments with the purpose to investigate removal of heavy metals from AMD sampled in Wales. The synthetic zeolites both showed the same selectivity for metal removal in the following decreasing order: Fe > As > Pb > Zn > Cu > Ni > Cr and the authors concluded these zeolites to be promising for the removal of heavy metals in AMD. Fly ash was converted to a synthetic zeolite by Prasad and Mortimer (2011). They performed batch experiments using AMD and could observe that an increased dosing with fly ash zeolites resulted in removal of 100 % Pb. The corresponding figures for Cd, Zn, Cu, Fe, Ni and Ba were 98.9, 98.8, 85.6, 82.8, 48.3 and 44.6 %, respectively. Prasad and Mortimer (2011) suggested retention on surface sites to be the main removal mechanism, due to the high cation exchange properties of the synthetic zeolites.

##### Summary

**Fig. 1:**
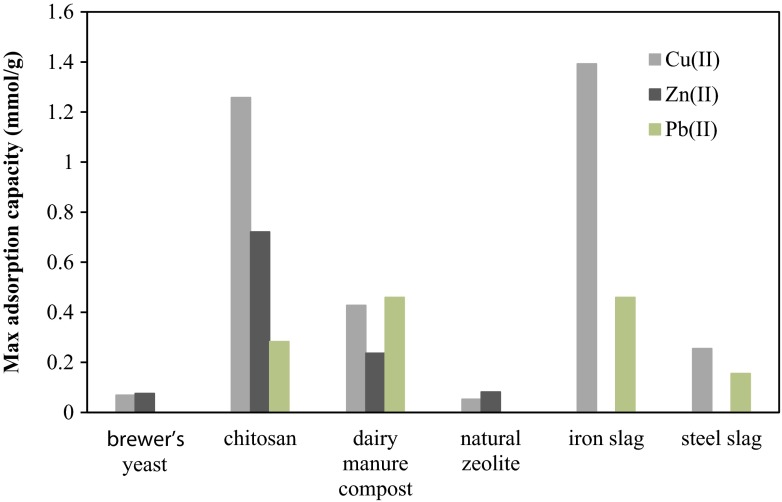
Comparison of the Langmuir maximum adsorption capacities defined for different filter materials. Experimental conditions are presented in Tables 2 and 3 (Ramirez-Paredes et al. 2011; Benavente et al. 2011; Zhang 2011; Feng et al. 2004; Motsi et al. 2009)

Various inorganic materials have been tested for removal of metals from mine drainage. Fly ash limestone, steel slag materials and zeolites have been investigated to the largest extent. Most of the experiments have been performed in laboratory, but some field experiments have also been reported. Results from laboratory experiments have shown promising metal retention for most of the materials tested under controlled laboratory conditions, while results from the field trials have been more various. Generally, it can be concluded that amongst the metals studied, Cu, Fe, Zn and Pb are easiest to remove by the filter materials such as steel and iron slag, lignite, chitosan, natural zeolite and yeast (Feng et al. 2004; Mohan and Chander 2006a; Laus et al. 2007; Mamba et al. 2009; Ramirez-Paredes et al. 2011). In the case of Fe, this has been attributed to the precipitation mechanism when removal of Fe is almost unaffected by the presence of other species in the solution (Motsi et al. 2009). For adsorption, a size and hydration tendency of different metals are strongly effecting on their uptake (Mamba et al. 2009). Chitin has been found to be a good adsorbent for Mn (Robinson-Lora and Brennan 2010b) and strong-base anion exchangers for U (Ladeira and Goncalves 2007). Figure 1 summarises maximum adsorption capacities of Cu, Zn and Pb obtained for different filter materials.

### Normalisation of data

A large number of filter materials have been investigated as a potential media for the removal of heavy metals from mining wastewaters as shown in Tables 2 and 3. Laboratory investigations have appeared to be the major experimental mode, but field trials under real life conditions have also been performed. The results presented in the tables show that some of the filter materials tested have performed well in both laboratory and field trials, which provides a certain basis for judgment of the filter materials potential for removal of metals even though the experiments have been carried out under laboratory conditions.

From the literature, it can be observed that the composition of the mining effluents vary to a large extent (Table 1) depending on the mining site and the type of mining. This is also one of the reasons why comparison of the results obtained by different research teams is challenging.

The literature review has also revealed that it is very hard to normalise the data on metal sorption reported from the different studies included in the review; thus, it is difficult to compare data. Several reasons for this can be mentioned. The first reason can be contributed to various physical and chemical properties of the filter materials tested in the different studies. For instance, pH, specific surface area and particle size have been reported to affect the capacity of metal removal.

#### Physical and chemical properties of filter materials

The pH of the filter material is highly important in the treatment of mining wastewaters. Especially, for acidic mine drainage the best filter materials for the metal removal are those with neutralisation capabilities. From materials presented in the literature fly ash, steel slag materials and chitin are observed to neutralise the solutions causing sulphate reduction and metal precipitation (Gitari et al. 2008; Feng et al. 2004; Pinto et al. 2011). Materials without neutralisation capability are not usually effective at acidic conditions due to the protonation of their surface groups.

The specific surface area of filter materials is another parameter of importance for metal removal and/or neutralisation. Not unexpectedly, Potgieter-Vermaak et al. (2006) reported an increased neutralisation rate when increasing the surface area for the pre-treatment agents studied, e.g. lime stone and dolomite. For bio-carbons, however, the metal removal from AMD samples was not dependent on the surface area, and it was stated that the feedstock and surface functionalities may play an important role as well (Shin et al. 2008).

Furthermore, the particle size of the filter material is important since it effects the hydraulic retention time. Filter materials with large particle size make the material highly permeable; thus, the metal solution or real-life mining waste-water will pass through the material quickly reducing the contact time. In field experiments described by Warrender et al. (2011), sand was mixed with the different filter materials used in the investigation in order to increase the permeability and maintain flow rates. A potential risk using filter materials with a small particle size is that clogging might occur.

#### Experimental conditions

The second reason is that there are no researchers who have carried out the experiments in the same way, and therefore, data should not be compared. Tables 2 and 3 show the range of experimental conditions that have prevailed in the investigations reviewed. It can, for instance, be seen that experimental conditions such as pH of metal solution/mine drainage, temperature, metal concentration and hydraulic retention time have varied, and these conditions are determining for the outcome of the research.

The temperature has also proved to be of importance for sorption/removal of metals. Mohan and Chander (2006a) demonstrated in batch tests performed at different temperatures, e.g. 10, 20 and 40 °C, that the removal efficiency varied between different metals with increasing temperature. Their results showed that Fe^2+^ was removed to a higher extent with increasing temperature while the opposite was demonstrated for Mn^2+^. In the first case, Mohan and Chander (2006a) suggested an endothermic process being responsible for the Fe^2+^–lignite system while an exothermic process being involved in the Mn^2+^–lignite system

pH is one of the most important parameters affecting metal removal by filter materials. This is because it affects the surface charge (protonation) of the solid material as well as speciation of dissolved components. In the literature reviewed, pH of the laboratory solution or the real-life mining wastewaters used have ranged between 2 and 9 (Table 1). However, as stated earlier, most of the filter materials applicable for the treatment of mining wastewaters have also neutralisation ability. For chitin metal uptake at low pH (<5) has been observed to be rather poor and almost total metal removal obtained at higher pH (>8) (Pinto et al. 2011; Robinson-Lora and Brennan 2010a). Improved efficiency of metal removal at higher pH has been assigned to the precipitation of metals. Treatment of AMD by fly ash has also increased the solution pH following increasing uptake of metals and reduction of SO_4_ concentration (Gitari et al. 2008).

The concentration of SO_4_^2−^ has also proved to be of importance for the metal uptake. Ladeira and Goncalves (2007) showed that the presence of SO_4_^2−^ had a negative effect on the uptake of uranium. This can be regarded as a drawback for the use of filter materials when the mine drainage has a low pH value.

The metal concentration is another factor affecting adsorption processes. By changing metal concentration and keeping other conditions (dose of filter material, contact time and temperature) constant, adsorption isotherms describing relations of adsorbed and free metals at equilibrium can be achieved. Robinson-Lora and Brennan (2010a) used Mn concentrations from 0.5 to 250 mg/L for isotherm studies with chitin and used the Langmuir and Freundlich equations for modelling. The Langmuir model gave better fitting and maximum adsorption capacity of 5.437 mg/g. Ramirez-Paredes et al. (2011) used both synthetic and real AMD and increased separately the concentration of Cu, Mn, Ni and Zn to obtain adsorption isotherms for *S. cerevisiae* yeast. Langmuir type behaviour was observed, but adsorption efficiency clearly decreased for Mn and Zn in real AMD samples while the removal of Cu and Ni was not influenced by the solution matrix. Zhang (2011) performed isotherm studies in which both single and multi-metal solutions as representatives of simulated AMDs were used. Data was described well with the Langmuir isotherm and at competitive conditions the adsorption efficiency of the three studied metals followed the order of Pb > Cu > Zn.

The hydraulic retention time has also proved to be of importance. Warrender et al. (2011) reported that filter materials such as compost, fly ash and iron ochre were mixed with sand in order to increase the permeability and maintain flow rates. These materials also became saturated with metals (Zn, Pb and Cd) rapidly, and this was attributed to the relatively high flow rates.

#### Experimental methods

The third reason for difficulties in normalisation of data can be referred to essential differences in experimental conditions that prevail in laboratory studies compared to those studies carried out in the field. It could be argued that data on sorption capacities should only be compared when similar experimental conditions have been employed. From Tables 2 and 3, it can be seen that the experimental conditions, which are determining the outcome of the investigations, have varied. Batch tests have been the main type of set-up in the laboratory according to the literature reviewed. The batch tests described have varied with regard to the amount of filter material/solution, metal solution(s) and metal concentration(s), pH, temperature and other factors as well. These differences make it difficult to compare results from different studies even though the same type of filter material has been used. In addition, batch tests have been criticised with regard to the potentially misleading data that they might result in. Drizo et al. (2002) argued that batch tests followed by calculations of the Langmuir adsorption isotherm might result in erroneous data since conditions in a batch test are far from those in the field.

Parameters such as temperature, pH, metal concentrations and hydraulic loading can easily be kept stable in the laboratory, but these are expected to vary during field conditions. Additional conditions occurring in field trials that will affect the results are precipitation and the composition of mine drainage used. This is especially true for constructions where the filter material is exposed to precipitation. The composition of acid mine drainage is far more complex than artificial metal solutions or artificial acid mine drainages prepared in the laboratory. The main reason for using these solutions in laboratory experiments is that they minimise influence of competitive ions for sorption and, in addition, minimise the influence of biological activity that could possibly disturb the physical and chemical sorption mechanisms involved in the removal of metals.

Based on the findings in the literature, it is difficult to suggest a standardised method for the investigation of the metal removal capacity of a filter material. Westholm (2006) faced the same challenge when describing removal of phosphorus using filter materials, and some researchers (Ádám et al. 2007) suggested standardised methods that could be used in the laboratory. It is possible that it would be feasible to suggest a standardised method also for the removal of metals in mine drainage by filter materials, at least if a laboratory method is to be defined. But this would not be as easy for field trials where local conditions such as character of mine drainage, weather conditions (e.g. temperature and precipitation) set the limit for what can be done. However, this is not within the scope of this paper.

The above-mentioned reasons could be brought forward as acceptable for difficulties in the normalisation of data. There are, however, other ways to judge if a filter material is suitable for metal removal or not and that is by the number and types of studies the filter material has been the subject of. A large number of studies, with different character, could, generally speaking, constitute a better basis for judgment of the filter material’s suitability for metal removal. In this survey, solely steel slag materials have been tested in the largest number of both laboratory-scale experiments as well as in field trials with promising results, and one might therefore be inclined to recommend these materials for further use.

#### Potential benefits for on-site treatment of acid mine drainage

Discharge of acid mine drainage is a vast problem world over, and the number of sites where treatment of acid mine drainage is needed is extensive. The interest for using filter materials is growing since the technique is promising, not only for acid mine drainage but also for other types of wastewater streams, for instance domestic wastewater, landfill leakage and storm water. The filter technique is regarded as an adequate alternative to more technical solutions or other small-scale solutions, and there are several reasons for this.

The literature review has showed that there are a large number of different filter materials that might be used for removal of heavy metals from acid mine drainage. This fact provides possibilities to use a local available material; thus, transports, etc. are not necessary at all locations. Using locally available filter materials is also advantageous due to low cost. The filter technique is also cheap compared to more high technical solutions since a smaller degree of maintenance is needed. In addition, it is not necessary to use precipitation chemicals as additives to promote the metal removal.

Table 4 presents a comparison of different adsorbents that have been used for the treatment of mining wastewaters.

**Table 4:** Comparison of different adsorbents used in the treatment of mining wastewaters

	Cost	Availability	Advantages	Disadvantages
Chitin	Low cost 0.8–31 Euros/kg (1–40 USD/kg)	Abundant, especially China and India	–Efficient removal of metals –Neutralising agent –Sulfate removal	–Variable composition –Swelling
Chitosan	12.2–230 Euros/kg (16–300 USD/kg)	Quite abundant, especially China, India, and Thailand	–Efficient removal of metals –Neutralising agent –Sulfate removal –Modification –Partial chemical regeneration	–Variable composition –Swelling –Soluble in dilute acids
Commercial ion-exchange resins	2–100 Euros/kg	Abundant	–Large variety of specific resins available –Chemical regeneration	–Different resins for anions and cations –High price in some cases –Swelling of polymeric resins –Loss of functionality during regeneration
Dairy manure compost	Low cost	Abundant	–Efficient removal of metals –Regeneration using acid	–Variable composition –Leaching of elements
Lignite	Low cost	Abundant	–Efficient removal of metals –Neutralising agent –Regeneration using acid	–Variable composition –Leaching of elements
Rice husk	Low cost	Abundant	–Efficient removal of metals –Regeneration using acid	–Variable composition
Yeasts	Low cost	Abundant	–Efficient removal of metals –Regeneration –Easy to modify	–Better in neutral conditions –Type of the wastewater has a significant effect
Commercial activated carbon	0.08–8 Euros/kg (0.1–10 USD/kg)	Abundant	–Known composition –Efficient removal of metals and organics	–Thermal regeneration –Poor adsorption of anionic species
Biocarbon	Low cost, depends on the source and treatment temperature	Abundant	–Efficient removal of metals and organics	–Thermal regeneration –Poor adsorption of anionic species
Fly ash	Low cost	Abundant	–Efficient removal of metals –Neutralising agent –Sulfate removal	–Variable composition –Leaching of elements
Furnace slag	Low cost	Abundant	–Efficient removal of metals –Neutralising agent	–Variable composition –Leaching of elements
Limestone	Low cost	Abundant	–Efficient removal of metals –Neutralising agent	–Formation of sludge as secondary waste
Natural zeolite	Low cost 0.04–1.9 Euros/kg (0.05–2.5 USD/kg)	Abundant, especially China, Indonesia, and Turkey	–Efficient removal of metals and anions –Modification	–Variable composition
Olivine	Low-cost	Abundant, especially China, India, and Turkey	–Efficient removal of metals	–Variable composition –Leaching of elements
Synthetic zeolite	Low cost 0.2–2.3 euro/kg (0.3–3 USD/kg)	Abundant, especially China, Indonesia, and Turkey	–Efficient removal of metals and anions –Modification	–Variable composition

Another advantage using filter materials is that treatment systems can achieve desired levels of metal attenuation regardless of site conditions. A treatment system based on filter materials can accumulate metals in a finite and accessible volume of filter material, thus making the material available for collection. This implies that the filter material is placed in the system in such a way that it will be easy to replace it when metal saturation is achieved.

#### Problems with on-site use of filter materials for removal of metals from mine drainage

From the literature reviewed, it is observed that only a few treatment facilities are in operation and since these are quite new, e.g. they have been in use for some years, the science is still young and immature and drawbacks have not been reported to a large extent. The use of filter materials for removal of metals from mine drainage might, however, be connected to some potential drawbacks. Based on the results in Table 4, one might, however, conclude that general disadvantages of using filter materials at large-scale applications are variable composition of mine drainage, high costs, instability, the potential leaching of hazardous substances from the filter materials and difficulties in regeneration. For low-cost filter materials, one alternative might be disposal at landfill sites unless other, more beneficial, uses can be found. Techniques for incineration of organic wastes from landfill sites and subsequent separation of metals are available today; thus, it might be possible to incinerate filter materials of organic origin and through various methods separate metals and transform them into commercial products. What to do with inorganic filter materials saturated with metals is a problem to be resolved in the future.

## Conclusions and future perspectives

A large number of different filter materials for potential use for removal of heavy metals from mine drainage have been reviewed. Most of these filter materials have been tested in laboratory experiments; others have also been investigated in field trials under real life conditions. The essential differences in experimental conditions that have prevailed in the experiments contribute to difficulties in normalisation of the results obtained. However, only a few filter materials have been included in tests of varying character, resulting in promising properties with regard to removal of heavy metals and in some cases, also provision of alkalinity. Field investigations have, for instance, demonstrated that steel slag materials remove metals, and these findings establish that the use of these materials contributes to a decrease in discharge of heavy metals to water bodies, however, to various extents. In addition, steel slag materials have also proved to provide alkalinity. Since the filter-based treatment method is a fledging one with regard to mine drainage, further research is suggested. Regeneration studies with metal-laden filter materials to recover the metals as well as filter materials should be conducted as well as further investigations of the importance of SO_4_^2−^ for the metal uptake. This will enhance the economic feasibility of the process. In addition, research should not be limited to laboratory-scale experiments, but column, pilot-scale and full-scale studies should also be conducted with different filter materials to investigate their potential on a commercial scale.
